# Cationic Peptides and Their Cu(II) and Ni(II) Complexes: Coordination and Biological Characteristics

**DOI:** 10.3390/ijms222112028

**Published:** 2021-11-06

**Authors:** Aleksandra Kotynia, Benita Wiatrak, Wojciech Kamysz, Damian Neubauer, Paulina Jawień, Aleksandra Marciniak

**Affiliations:** 1 Department of Inorganic Chemistry, Wroclaw Medical University, Borowska 211A, 50-556 Wroclaw, Poland; 2 Department of Pharmacology, Wroclaw Medical University, Jana Mikulicza-Radeckiego 2, 50-345 Wroclaw, Poland; benita.wiatrak@umw.edu.pl (B.W.); paulina.jawien@umw.edu.pl (P.J.); 3 Department of Inorganic Chemistry, Medical University of Gdańsk, Gen. J. Hallera 107, 80-416 Gdańsk, Poland; wojciech.kamysz@gumed.edu.pl (W.K.); damian.neubauer@gumed.edu.pl (D.N.); 4 Department of Biostructure and Animal Physiology, Wroclaw University of Environmental and Life Sciences, Norwida 25/27, 50-375 Wroclaw, Poland

**Keywords:** cationic peptides, Cu(II) complexes, Ni(II) complexes, anti-inflammatory, potentiometric titration, spectroscopic methods, LPS-neutralization

## Abstract

Antimicrobial peptides are a promising group of compounds used for the treatment of infections. In some cases, metal ions are essential to activate these molecules. Examples of metalloantibiotics are, for instance, bleomycin and dermcidin. This study is focused on three new pseudopeptides with potential biological activity. The coordination behavior of all ligands with Cu(II) and Ni(II) ions has been examined. Various analytical methods such as potentiometric titration, UV-Vis and CD spectroscopies, and mass spectrometry were used. All compounds are convenient chelators for metal ion-binding. Two of the ligands tested have histidine residues. Surprisingly, imidazole nitrogen is not involved in the coordination of the metal ion. The *N*-terminal amino group, Dab side chains, and amide nitrogen atoms of the peptide bonds coordinated Cu(II) and Ni(II) in all the complexes formed. The cytotoxicity of three pseudopeptides and their complexes was evaluated. Moreover, their other model allowed for assessing the attenuation of LPS-induced cytotoxicity and anti-inflammatory activities were also evaluated, the results of which revealed to be very promising.

## 1. Introduction

Centuries ago, in traditional medicine, it was noticed that wearing jewelry containing metals such as copper, iron, and silver exhibited antimicrobial properties. Nowadays, drug-resistant pathogens are becoming more common. There is a risk that in the future there will be no cure for bacterial infections. Therefore, new treatments are being sought. From this point of view, antimicrobial peptides (AMPs) seem to be attractive and promising tools in medical sciences. Additionally, many metallo-complexes characterized in recent years have been applied in the treatment of anti-inflammatory infections, cancer, and neurodegenerative diseases [1,2,3,4].

Antimicrobial peptides constitute one of the basic and primitive defense mechanisms of living organisms of both plants and animals. They show a wide spectrum of antibacterial, antiviral, antifungal, anticancer, and antioxidant activities. Hence, AMPs could be potentially useful in medicine [5,6,7]. So far, over 4000 peptides have been identified in the database of The Data Repository of Antimicrobial Peptides (DRAMP) [8]. Five subgroups can be distinguished in the AMPs: anionic peptides; short helical cationic peptides without cysteine residues; peptides rich in amino acids such as proline, arginine, phenylalanine, or tryptophan; and anionic as well as cationic peptides containing disulfide bonds capable of forming stable β-sheets [9].

Metal ions are coordinated by more than 25% of the proteins in cells [10]. The metal ions are associated with the prosthetic groups of enzymes and play a key role in their activity. Proteins containing divalent d-block metal ions participate in many biological redox processes. In proteins, metal cations are usually bound either by nitrogen, oxygen, or sulfur atoms of the peptide side chain [11]. The *N*-terminal amine (-NH_2_) and *N*-amides (N^−^−C=O) of the peptide bonds, as well as imidazole nitrogen atoms (N_π_) from histidine residues, are the main anchoring groups for Cu(II) and Ni(II) ions [12,13].

Coordination with a metal ion is also essential to ensure the activity of antimicrobial peptides. Metal ions play an important role in the activity of AMPs and in their interaction with other biomolecules, e.g., proteins or nucleic acids. Here, the maintenance of a proper structure of molecules in the presence of a metal ion is often of vital importance [14]. An example of such a compound is bacitracin, a cyclic peptide synthesized by *Bacillus licheniformis* [5]. Its affinity to divalent metal ions decreases in the following order: Cu(II) > Ni(II) > Co(II), Zn(II) > Mn(II). It forms 1:1 complexes with metal ions [15,16]. Coordination characteristics of bacitracin with Zn(II) and Ni(II) were studied by potentiometric titration and UV-Vis spectroscopy by Scogin and co-workers in 1980 [17]. The authors have shown that the metal directly coordinates to the glutamate carboxyl, the histidine imidazole, and the thiazoline ring. These results eliminated the previously reported metal binding properties of this peptide [17]. Another example of interesting metal–peptide complexes is the Mn(II)-bacitracin complex and its SOD activity. Piacham and co-workers demonstrated its potential usefulness as an effective agent against oxidative stress [18].

Another example of metallo-antibiotics is bleomycin. It was first isolated from *Streptomyces verticillus* as a blue powder containing copper(II) [19]. It also forms coordination compounds with Mn(II), Fe(II/III), Co(II/III), Ni(II/III), Zn(II), Cd(II), Ga(III), and Ru(II) [14]. Imidazole, pyrimidine, amines of β-aminoalanine, and the amide nitrogen of β-hydroxyhistidine are donors for the metal ion in the case of the Cu(II) complex [20]. Dermcidin, an AMP from human sweat, requires Zn(II) ions for its activation. The metal ion induces and stabilizes the oligomerization of self-assembly complexes which form channels in the bacterial membrane [21]. The Cu(II) complexes with piscidins, linear, cationic, and amphipathic peptide families, isolated from the hybrid striped bass, exhibited enhanced bactericidal effects by inducing oxygen-free radicals as well as those cleaving DNA [22]. The coordination behavior of piscidins toward Cu(II) and Ni(II) ions was intensely studied and revealed a binding-like ATCUN motif with {NH_2_, 2 × N^−^,N_His_} coordination mode [23].

The cationic peptides can improve antimicrobial activity by electrostatic interactions with the negatively charged bacterial membranes [24,25,26]. The non-proteinogenic amino acids, such as Orn and Dab, are lysine analogs enhancing protease stability and proved a way to design new AMPs [27].

Many metal-based agents are characterized by interesting biological activities and, due to this, they show the possibility of application in medicine and pharmacy. Among them, a large group of compounds concerns copper complexes. In addition to peptides, many types of molecules can be coordinated with Cu(II) ions. This group includes thiosemicarbazone derivatives [28], catechol-containing Schiff bases [29], or substituted benzoic acids [30]. Many of the listed derivatives have anticancer activity. Therefore, copper complexes seem to be very attractive in the medicinal chemistry area.

**Figure 1:**
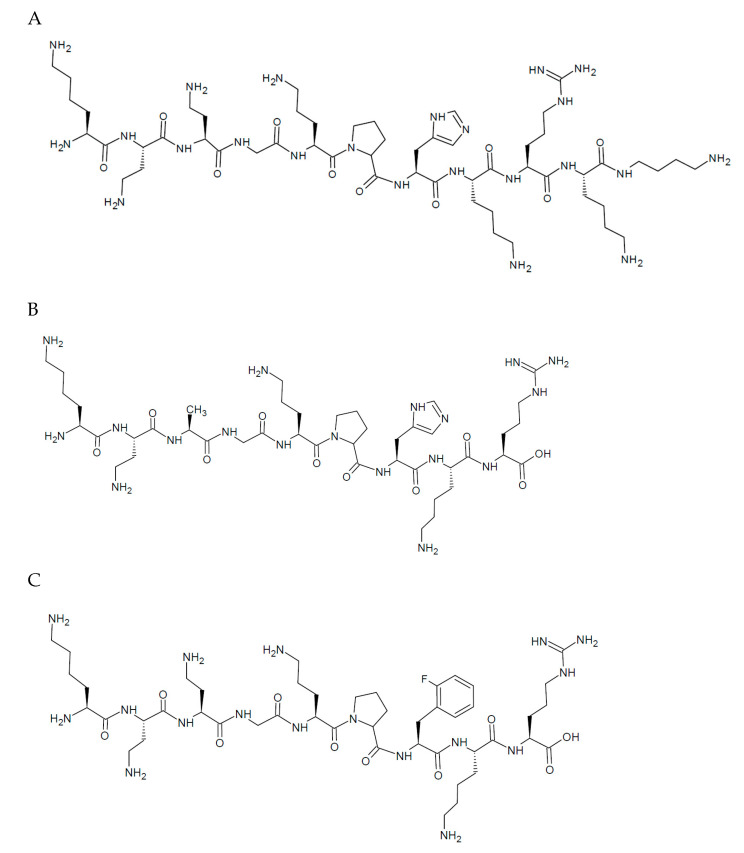
Amino acid structures of the ligands: (**A**) L1 (Lys-Dab-Dab-Gly-Orn-Pro-His-Lys-Arg-Lys-Dbt), (**B**) L2 (Lys-Dab-Ala-Gly-Orn-Pro-His-Lys-Arg), and (**C**) L3 (Lys-Dab-Dab-Gly-Orn-Pro-Phe(2-F)-Lys-Arg).

This study is focused on three novel peptides, namely L1, L2, and L3, whose sequences and structures are presented in Figure 1. They do not have blocked terminal units (-NH_2_ and -COOH terminals) and are rich in amino groups belonging to peptide side chains, which contribute to the cationic nature of the molecules. Moreover, the coordination of metal ions by the compounds increases the net charge of the molecule. The presence of the Orn and Dab residues in the sequence can also contribute to the enhancement of the proteolytic stability of the compounds and their anti-mycobacterial activities [27]. To summarize, it can be stated that this study explores the synthesis of peptides, evaluates the coordination properties to Cu(II) and Ni(II) metal ions, and evaluates their biological activity.

## 2. Results and Discussion

### 2.1. Acid/Base Properties of the Compounds

The fitting of the potentiometric titration curves enabled the determination of the protonation constants for the ligands. The obtained results are listed in Table 1. L1 shows ten protonation groups, whereas L2 and L3 have eight. The most acidic values, log*K* 2.50 and 2.64, belong to the carboxyl C-terminal group of L2 and L3, respectively.

The values seemed to be quite low, but in proteins, with a positive charge, the log*K_COO_* tended to be lower than those in the relative uncharged protein model [31,32]. These ligands possessed a high positive charge in an acidic environment due to the presence of few amino (-NH_2_) groups in amino acid side chains. Moreover, similar values of log*K_COO_* were found previously in peptides with unprotected C-termini [33,34,35,36]. The protonation steps for the histidine side chains fell in the pH range between 5 and 7, and the log*K_His_* values were 5.86 and 5.96 for L1 and L2, respectively. They were similar to those characteristic (N_π_) in the imidazole ring where the average log*K_His_* is 6.6 [31]. However, in many models of peptide ligands, lower values were reported [37,38,39,40,41]. A second (N_τ_) imidazole nitrogen remains protonated and, usually, proton dissociation occurs, here, at high pH and log*K* = 14 [42]. Further dissociation processes belong to amino groups from the *N*-terminal, side chains of Dab and Orn, and Lys residues. The investigated ligands were rich in -NH_2_ groups, thus the protonation reactions were overlapping processes and the stepwise constants were very similar to each other. These are the reasons why it was impossible to precisely indicate which log*K* belonged to which specific amino group. However, it could be expected that pKa values increase with the number of methylene groups in the side chain (Dab < Orn < Lys), as reported in the previous work by Bruckres et al. [43]. The values of protonation constants (log*K*) for these reactions fall in the range of 9.5–10.7 [43,44,45,46]. The protonation constants for HL species for all three ligands were the highest and appeared in an alkaline environment. This reaction could be assigned to the guanidine group of the side chain of Arg residue.

### 2.2. Interaction with Copper (II) Ions

**Figure 2:**
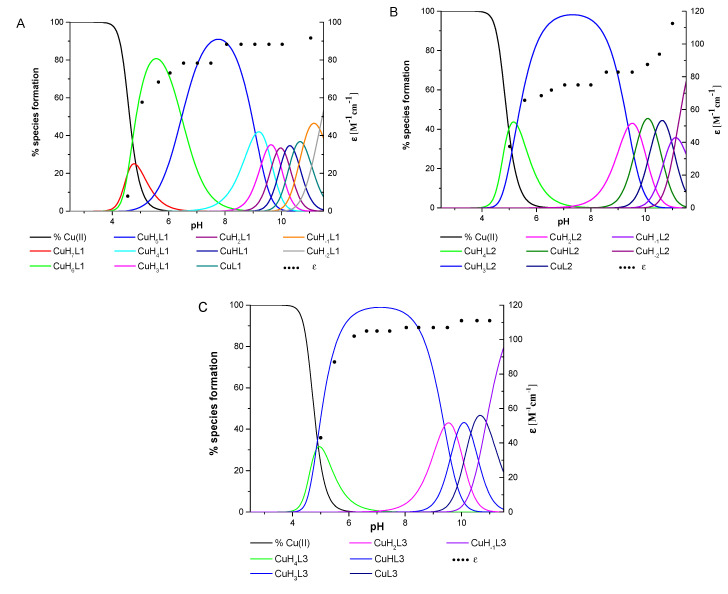
The species distribution diagrams of systems (**A**) L1:Cu(II), (**B**) L2:Cu(II), and (**C**) L3:Cu(II) as a function of pH, together with the corresponding absorption coefficients (black dots).

**Figure 3:**
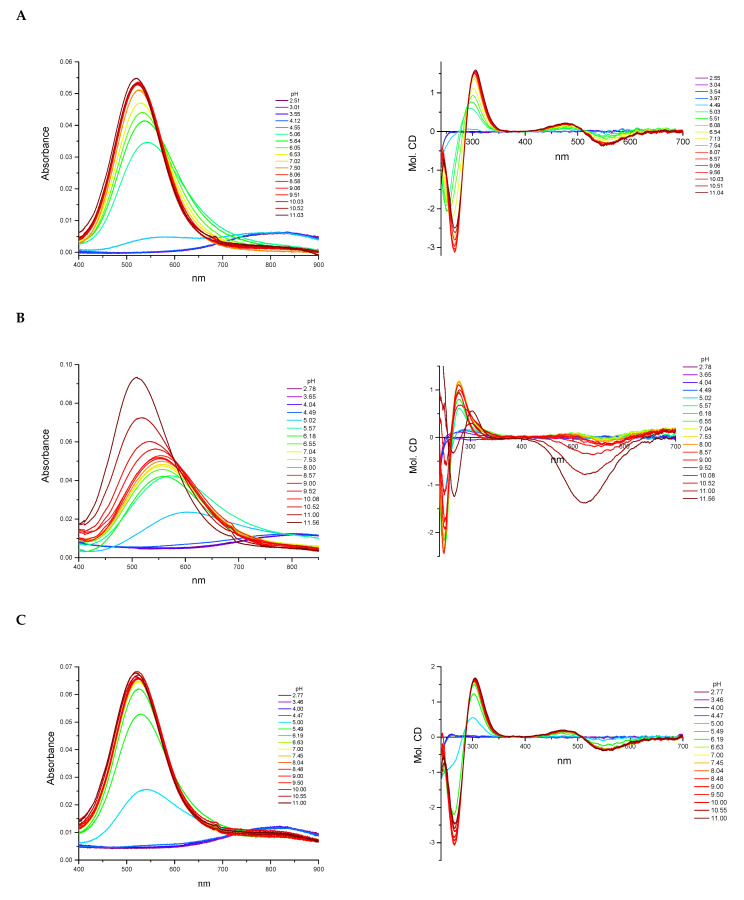
The UV-Vis and CD spectra at different pH values for the analyzed peptides with Cu(II) ions: (**A**) L1:Cu(II) system, (**B**) L2:Cu(II) system, and (**C**) L3:Cu(II) system.

All three ligands began the coordination process with Cu(II) ions at pH 4.0 and created only mononuclear species over the studied pH range. The percentage formation of species in equimolar ligand-to-metal ion ratio systems is presented in Figure 2. The overall stability constants and the spectroscopic data for complex species are listed in Table 2 and Tables S7–S9 and Figure 3.

**Figure 4:**
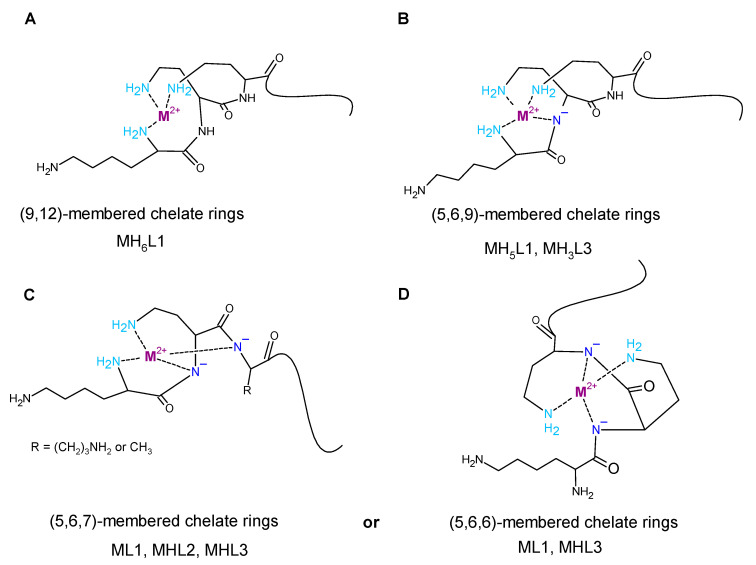
Proposed structures of the major complexes with following donor atoms set: (**A**) {NH_2 (*N*-terminal)_, 2 × NH_2 (Dab)_}, (**B**) {NH_2 (*N*-terminal)_, 2 × NH_2 (Dab)_, N^−^_(peptide bond)_}, (**C**) {NH_2 (*N*-terminal)_, NH_2 (Dab)_, 2 × N^−^_(peptide bond)_}, and (**D**) {2 × NH_2 (Dab)_, 2 × N^−^_(peptide bond)_} where M(II) = Cu(II) or Ni(II).

**Figure 5:**
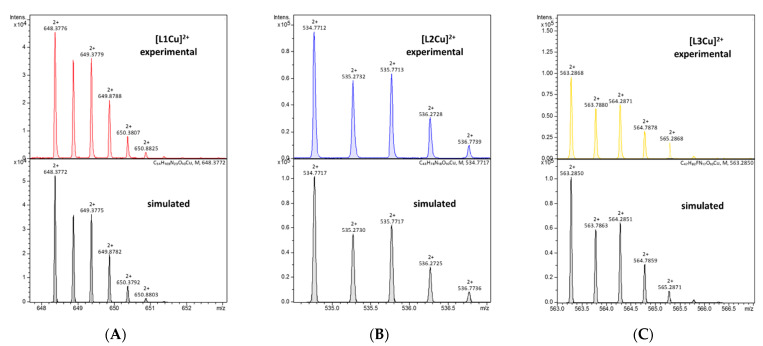
Experimental and simulated ESI mass spectra for (**A**) L1, (**B**) L2, and (**C**) L3 with Cu(II) ions in an aqueous solution at pH 7.0.

The L1 ligand began the coordination process via the creation of the CuH_7_L1 form, which arose simultaneously with CuH_6_L1 species. At pH 5.5, the CuH_6_L1 achieved a maximum concentration. The absorption band at 543 nm suggests the three nitrogen atoms involved in Cu(II) ions’ coordination manner (Figure 4A and Table S7). Moreover, the negative signal in the CD spectrum at λ = 252 nm and the positive signal at λ = 297 nm indicate that the donor atoms originated from the amino groups (Figure 4A and Table S7). This finding indicates that the *N*-terminal amino group became an anchoring center for the metal ion. Furthermore, both side chains of neighboring Dab residues were also the donors for the metal ion and complex in an {NH_2 (*N*-terminal)_, 2 × NH_2 (Dab)_} coordination mode to form a 9 and 12 mer chelate ring (Figure 4A). In the solution, between pH 7.0 and 8.5, the predominant CuH_5_L1 form appeared in the system. The ESI-MS spectra show a peak at *m/z* [648]^2+^, which has an isotope pattern typical of the Cu(II) complex and as confirmed by simulation (Figure 5A). The blue shift of the band to 524 nm with increasing intensity indicates the appearance of a next nitrogen atom in the Cu(II) coordination sphere (Figure 3A and Table S7. The CD spectrum confirms the participation of the amine group and also a contribution of the amide nitrogen. The negative (λ = 252nm → λ = 264nm) and positive (λ = 297nm → λ = 303nm) bands were shifted only slightly. Additionally, the Cotton effect for the d–d transition was noticed. Besides, the log*K* = 9.09 is a typical value of bounding Cu(II) via amide nitrogen from the peptide bond and supported the formation of the 4N complex. The proposed structure of CuH_5_L1 was based on the formation of a 5, 6, and 9 mer chelate ring, as shown in Figure 4B.

At a higher pH, several species coexisted at equilibrium in solution but the spectroscopic behavior was similar up to a pH of 10.5. Despite the variable overall charge of the complexes, the donor atoms of the binding metal ions remained the same: {NH_2 (*N*-terminal)_, 2 × NH_2 (Dab)_, N^−^_(peptide bond)_}. Some subsequent deprotonation steps accounted for the proton losses by the side chains of Dab, Lys, Orn, and Dbt. Above pH 10.5, an increase in the absorption coefficient (ε = 91.6 M^−1^cm^−1^) shifted the maximum by 5 nm to shorter wavelengths. Presumably, the second amide nitrogen appeared in the equatorial plane of the Cu(II) ions, replacing one of the -NH_2_ groups of Dab or the *N*-terminal. Replacement of the amino group of the Dab side chain would result in 5, 6, and 7 chelate ring-sizes (Figure 4C). In the case of *N*-terminal group replacement, the 5, 5, and 6-membered ring would be formed (Figure 4D). The second option seems to be more probable due to the higher thermodynamic stability [47]. This is compatible with the CD spectrum recorded at pH 11.0, where Δε_(λ__266)_ = −2.47 and the charge transfer of the NH_2_→Cu(II) transition band became weaker (Table S7). Therefore, in the final complex form, the metal ion was coordinated by four nitrogen donors: {NH_2 (*N*-terminal)_, NH_2 (Dab)_, 2 × N^−^_(peptide bond)_} (Figure 4D).

The Cu(II) coordination process of L2 initiated via the formation of CuH_4_L2. Since a fully protonated L2 ligand has eight stability constants, this CuH_4_L2 form dissociated four of them. According to the L2 stability constants (Table 1), they could be COO^−^, the imidazole side chain of His, and two of the amino group: the *N*-terminal and the side chain of the Dab residue. However, the spectroscopic data relates to a mixture of a copper aqua complex (Cu(H_2_O)_6_^2+^) and another complex form, CuH_3_L2, that coexisted in the solution. This excluded the possibility of unambiguity, suggesting particular donor atoms of the metal ions. The next species, CuH_3_L2, was well separated in the pH range of 6.5 to 7.5. In the ESI-MS spectrum, there was the [534]^2+^ *m/z* signal with the typical isotopic pattern for the Cu(II) complex with L2 ligand (Figure 5B). The location of the maximum absorption at 557 nm affirmed three nitrogen donors involved in binding Cu(II) ions. The CT bands in the CD spectrum, specifically a positive band at 277 nm and a negative band at 249 nm (Figure 3B and Table S8), suggested interaction of the metal ion with the -NH_2_ group and amide nitrogen from the peptide bond, respectively. The *N*-terminal amino group also seemed to be an anchoring center and the coordination mode for CuH_3_L2 could be identified as {NH_2 (*N*-terminal)_, NH_2 (Dab)_, N^−^_(peptide bond)_}. The previous study on β-amyloid fragments confirmed that the *N*-terminal amino group and imidazole ring are competing for interactions with metal ions [48]. Especially in the physiological pH range, the coordination process starting from the *N*-terminal is more preferable to the side chain of His residue, which is located far from the -NH_2_ termini group [47,48].

An increasing pH triggered further deprotonation steps, which are likely to be related to the dissociation of the side chains of Lys and Orn residues because the spectroscopic profiles did not change up to a pH of 10.0. Above this value, both the significant shift of the absorption band to 509 nm and the simultaneous increase in the extinction coefficient indicated involvement of subsequent nitrogen in the binding of Cu(II) ions. Moreover, a large variation in the CD spectrum was also noticed (Figure 3B). The NH_2_→Cu(II) CT peak moved to 303 nm, the N^−^→Cu(II) CT signal shifted to 268 nm, and the negative Cotton effect (λ = 529 nm) due to the d–d transition band was observed (Table S8). The similarity of the spectra recorded at high pH for the ligands L1 and L2 indicates that the final complex form was characterized by the same donor atoms in the environment of the Cu(II) ions. The coordination mode of the final complex form can be described as {NH_2 (*N*-terminal)_, NH_2 (Dab)_, 2 × N^−^_(peptide bond)_} (Figure 4C).

The spectroscopic data recorded for L3 with Cu(II) ions were almost identical with those of L1 (Table S9 and Figure 3C, respectively), thus indicating similarities of the coordination processes for these two compounds. The L3 ligand began to bind to the Cu(II) ions via the formation of CuH_4_L3. This species coexisted in solution with the aqua complex of copper ions (Cu(H_2_O)_6_^2+^) and CuH_3_L3. Hence, it was impossible to unambiguously identify the donor atoms. The concentration of CuH_3_L3 increased upon rising the pH and that form dominated in the solution at a pH between 5.5 and 8.5. The formation process of CuH_3_L3 resembled that of CuH_5_L1. The 525 nm band and CD spectroscopic data (Δε_302_ = 1.49, Δε_265_ = −2.80, Table S4) were comparable to those of CuH_5_L1 and suggest the coordination mode: {NH_2 (*N*-terminal)_, 2 × NH_2 (Dab)_, N^−^_(peptide bond)_} (Table S9). Moreover, in the ESI-MS spectra, the *m/z* [563]^2+^ peak confirmed the presence of CuH_3_L3 and was compatible with simulation (Figure 5C). The appearance of the next CuH_2_L3 species did not follow any spectral variations. This suggests deprotonation of the ligand molecule without impact on the metal ions’ coordination sphere. At a pH above 10.0, the maximum absorption moved slightly to higher energy wavelengths (λ_max_ = 520 nm, Table S9 due to the formation of CuHL3. There is strong evidence of metal ions binding to 4N donor atoms. The CD molar coefficient of the negative signal at 266 nm was tends towards smaller values (Table S9). This confirms the replacement of one -NH_2_ group on N^−^_amide_ in the coordination sphere of Cu(II) ions. Based on these observations, it can be concluded that CuHL3 exhibits the {2 × NH_2 (Dab)_, 2 × N^−^_(peptide bond)_} coordination mode, characterized by the participation of the same donor atoms both in CuH_-1_L1 and CuH_-2_L1 (Figure 4D). Finally, the last two protons dissociated in the studied system. The log*K* values, specifically 10.33 and 10.88, corresponding to the formation of the CuL3 and CuH_-1_L3 species, respectively, and are typical of the amino side chain of Lys residues. These constants are comparable with those of log*K*(H_3_L3) and log*K*(H_2_L3) (Table 1). The formation of subsequent coordination complexes had no impact on the spectroscopic data, thus suggesting that the donor atoms did not change in the metal ion sphere.

**Figure 6:**
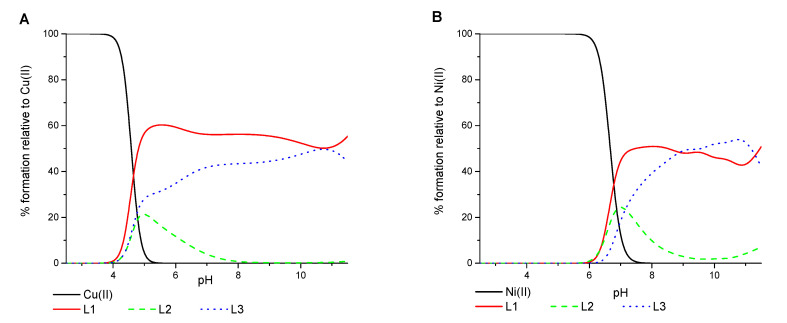
Competition graph of the efficiency of the binding metal ions by compounds L1 (solid red line), L2 (dashed green line), and L3 (dotted blue line) as a function of pH for: (**A**) Cu(II)/L and (**B**) Ni(II)/L.

A comparison of the efficiency in Cu(II) ions binding between the ligands shows that L1 formed the most stable complex over the whole pH range (Figure 6A). L3 has a comparable but slightly weaker behavior. L2 revealed to be the least competitive one. This finding is probably because the *N*-terminal peptide region in both L1 and L3 is rich in anchoring amino groups also from the side chain of the Dab residue, which are involved in metal ion binding.

### 2.3. Interaction with Nickel (II) Ions

**Figure 7:**
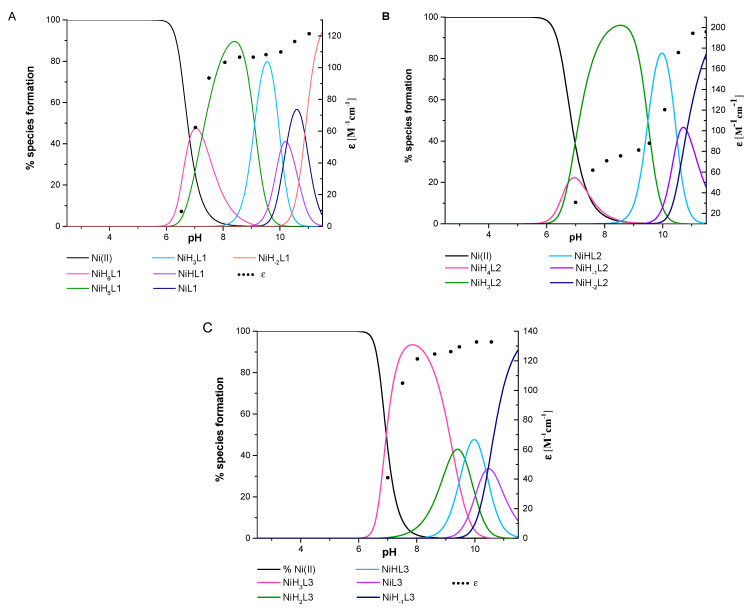
The species distribution diagrams of systems (**A**) L1:Ni(II), (**B**) L2:Ni(II), and (**C**) L3:Ni(II) as a function of pH, together with the corresponding absorption coefficients (black dots).

**Figure 8:**
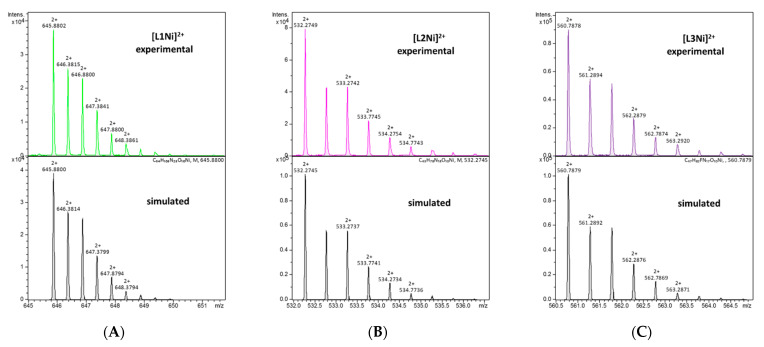
Experimental and simulated ESI mass spectra for (**A**) L1, (**B**) L2, and (**C**) L3 with Ni(II) ions in an aqueous solution at pH 7.0.

All three ligands began to bind Ni(II) ions above pH 6.0. The stability constants and stepwise constants are presented in Table 3, and complex forms of the distribution diagrams for each Ni(II)/L system are shown in Figure 7. The ESI-MS spectra exhibited only mononuclear signals, specifically *m/z* = [645]^2+^ for NiL1, *m/z* = [532]^2+^ for NiL2, and *m/z* = [560]^2+^ for NiL3, confirming the stoichiometry of the species obtained by the potentiometric titration (Figure 8).

**Figure 9:**
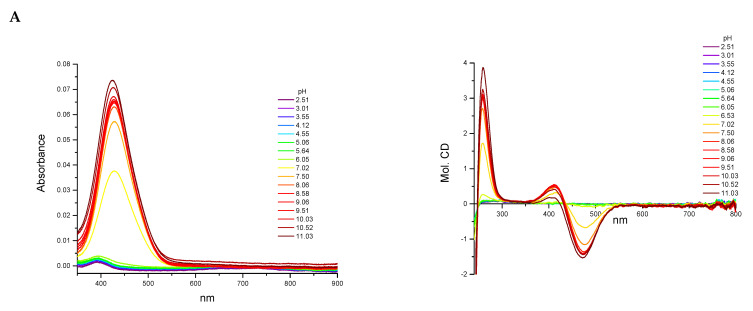

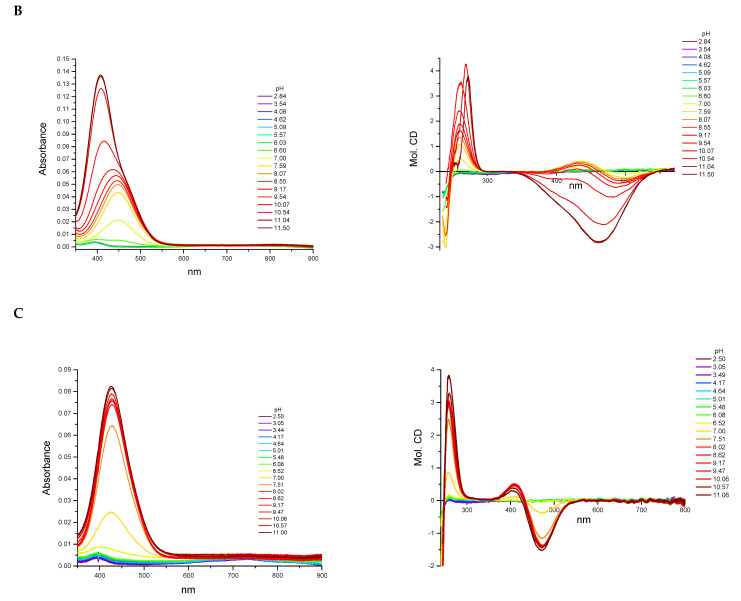
The UV-Vis and CD spectra at different pH values for the analyzed peptides with Ni(II) ions: (**A**) L1:Ni(II) system, (**B**) L2:Ni(II) system, and (**C**) L3:Ni(II) system.

The starting point for the coordination of L1 with Ni(II) ions marked the appearance of NiH_6_L1 but a distinct spectroscopic signal occurred above pH 7.0, where NiH_5_L1 became prevalent in the system. This species lacks five protons, thus, according to log*β* and acid-base specification, this could suggest the presence of a side chain of His, both the Dab and *N*-terminal amino group, and probably one of the amide groups of the peptide bond. The spectral band around 429 nm supported the presence of 4N donors (Table S10 and Figure 9A). This is compatible with the previously studied tetrapeptides with the ATCUN motif and their cyclic analog [49], specifically dehydrotripeptides: Gly-ΔAla-Phe, Gly-(E)ΔPhe-Gly [50], or even multihistidine peptide Ac-HAAHAAHAAHAAHAAHAAH-NH_2_ [51]. Moreover, the CT→N^−^ 258 nm band together with the negative (Δε_477_ = −1.35) and the positive (Δε_417_ = 0.51) d–d bands (Table S10) support the thesis that the following four nitrogen atom donors participate in the complex formation: {NH_2 (*N*-terminal)_, 2 × NH_2 (Dab)_, N^−^_(peptide bond)_}. Upon raising the pH, subsequent deprotonation steps occurred in the system and both NiH_3_L1 and NiHL1 were formed. However, spectroscopic parameters remained invariant up to pH 10.0, thus suggesting that the coordination donor atoms remained invariant and dissociation reactions were associated with the amine groups of the side chains of the Orn, Lys, and Dbt residues. Above pH 10, a bluish shift of the absorption band was observed with an increase in the molar extinction coefficient. Additionally, the CT→N^−^ signal (258 nm) became intense (Table S10). This is probably associated with the exchange of one of the Dab amino groups in the nickel ions coordination sphere by another amide nitrogen to create a stable square–planar complex with 5, 6, and 6-membered chelate rings. This complex is more thermodynamically stable than that of the previous coordination mode (Figure 4D). The appearance of the final species, NiH_-2_L1, is probably related to the deprotonation of the guanidine group of the Arg residue, which usually occurs at a high pH.

The concentration of the first Ni(II) species of the L2 compound, NiH_4_L2, was negligible and coexisted with free metal ions as well as with the next NiH_3_L2 form in the solution. Hence, it was impossible to define a coordination mode for the first complex. The d–d transition band at 450 nm detected distinct evidence for the square–planar complex with a three nitrogen donor set for NiH_3_L2 (Figure 9B, Table S11) [52]. Upon raising the pH, the extinction coefficient intensified, although the signal position remained unchanged, suggesting the dominance of the same coordination donor atom up to a pH of 9.0 (Table S11). The CD spectra indicated the Ni(II) ion coordination by amino groups (CT→NH_2_ negative band at 239 nm and amide nitrogen CT→N^−^ positive band at 260 nm, Table S11 and Figure 9B). The logK^*^ = log*β*_NiH3L_−log*β*_H3L_ = 8.65 supported the metal ion-binding via the amide nitrogen of the peptide bond. Accordingly, the most probably donor atom set for NiH_3_L2 is {NH_2 (*N*-terminal)_, NH_2 (Dab)_, N^−^_(peptide bond)_}. Above pH 9, when NiHL2 emerged, a hypsochromic shift effect to 435 nm in the maximum absorption was observed. Simultaneously, a stronger 260 nm band in the CD spectrum (Table S11) indicated the participation of the next amide nitrogen donor in the metal ion bounding. A further shift of the band to shorter wavelengths (416 nm) occurred above pH 10 and supported the existence of four nitrogen donors surrounding the Ni(II) ions. The large variation in the CD spectra, especially the peak at 268 nm (Table S6), implies the replacement of one amino group by the third amide nitrogen of the peptide chain. Thus, the proposed coordination mode for NiH_-1_L2 could be {NH_2_, 3N^−^_(peptide bond)_}. Consecutive NiH_-2_L2 and NiH_-3_L2 forms were related to the dissociation of the next two hydrogen atoms from the Lys and Arg residues, respectively.

The spectroscopic behavior of L1 and L3 with Ni(II) ions was alike and similar to systems with copper(II) ions. The first complex with NiH_3_L3 stoichiometry dominated over a wide pH range and attained a maximum concentration at pH 7.8. The UV-Vis absorption spectrum was characterized by a well-defined band at 428 nm (Table S12 and Figure 9C). The CD parameters for the d–d transition band were Δε_473_ = −1.36, Δε_409_ = 0.50, and CT→N^−^: Δε_258_ = −2.89 (Table S12), and were related to donor atoms identical to NiH_5_L1, i.e., {NH_2 (*N*-terminal)_, 2 × NH_2(Dab)_, N^−^_(peptide bond)_} (Figure 4B). The formation of subsequent complexes at higher pH, caused by the deprotonation of the Orn and Lys amino side chain groups, proceeded without affecting spectroscopic data (Figure 9C) and thus the coordination sphere of the metal ions remained unchanged. Around pH 10, there were small variations in the spectroscopic parameters. Presumably, they were caused by the replacement of the NH_2 (Dab)_ group by the amide nitrogen N^−^ of the peptide backbone. The resulting complex form was still of the 4N coordination type. For this ligand, dissociation from the guanidine Arg group was missing.

The simulation of competitive Ni(II) ions binding via the three ligands studied is shown in Figure 6B. Between pH 6 and 9, L1 dominated the Ni(II) ions binding, but L3 competed with it to form more stable complexes over the pH basic range above 9.0. However, the L1 and L3 ligands showed comparable coordination behavior during Ni(II) ions binding.

### 2.4. Biological Evaluation

**Figure 10:**
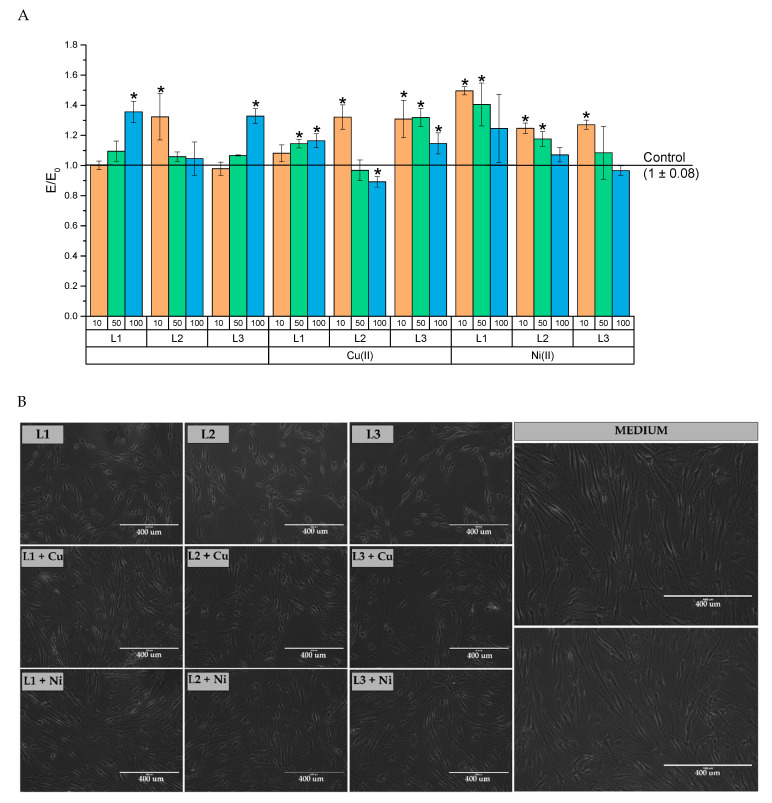
(**A**) Evaluation of the cytotoxicity of compounds in the MTT assay: control—cell culture incubated without test substances and * *p* < 0.05—significant difference compared to the negative control. (**B**) Cell morphology for the NHDF line after treatment with 100 µM of the test compounds or complexes and without test substances (white line scale–400 um).

The percentage of viable NHDF cells after exposition to the tested compounds and their complexes with Cu(II) or Ni(II) ions was compared with the viability of the cells incubated in a culture medium only (100% viability). The results are shown in Figure 10. In all test concentrations after contact with the test solutions, the number of metabolically active cells did not drop below 70% (Figure 10A). After 24 h of incubation, the viability level reached no less than 89%, which was only this low after using the copper complex (CuH_3_L2) at a concentration of 100 µM. An increase in cell viability was also noticed (on average, about 135%) at a concentration of 100 µM for compounds L1 and L3, and at 10 µM for compound L2. A statistically significant increase in mitochondrial activity was also found when using complexes of NiH_3_L1 and NiH_3_L2 in concentrations of 10 and 50 µM, as well as NiH_3_L3 only in 10 µM concentrations. On the other hand, complexing compounds with Cu(II) caused statistically significant enhanced mitochondrial activity over the entire concentration range of compound L3 and over a range of 50–100 µM for compound L1, whereas this only occurred at a concentration of 10 µM for compound L2. No inhibition of cell viability below 70% indicates the lack of cytotoxic potential of both these compounds and their metal complexes.

No cell lysis or growth inhibition was noticed during the microscopic evaluation of the cell cultures treated with the compounds and their complexes for 24 h. A single cytoplasmic granulation was observed only. Cell morphology was normal for the NHDF cell line (Figure 10B).

#### 2.4.1. Study on Attenuation of LPS-Induced Cytotoxicity

**Figure 11:**
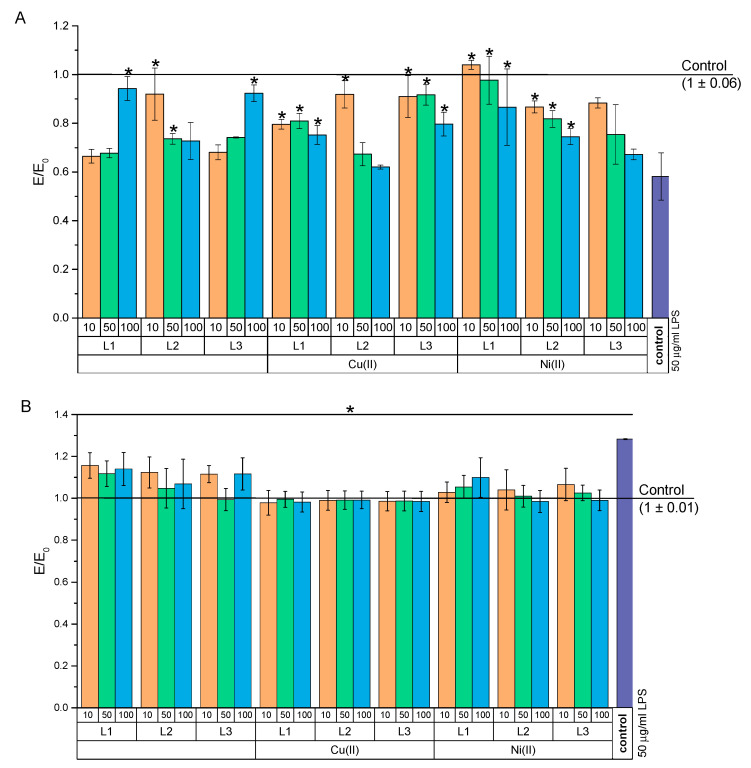
Effect of test compounds on NHDF cell culture incubated with LPS: (**A**) MTT test and (**B**) DCF-DA test. control—cell culture incubated without LPS and test substances and * *p* < 0.05—significant difference compared to the control preincubated with LPS.

Due to the increased mitochondrial activity of NHDF after 24 h of incubation with the test compounds (indicating the plausible effect of these compounds on the growth of proliferation), we decided to check the effect of the test compounds and their complexes with Cu(II) or Ni(II) ions on the damage caused by endogenous bacterial fragments and lipopolysaccharides (LPS) (Figure 11A). In this study, the viability of NHDF after a 24 h incubation with 50 µg/mL of LPS was only 58% compared to that of the negative control. At the same time, treating the cells with test compounds improved the activity of NHDF mitochondria. The strongest growth was demonstrated in a cell culture treated with 10 µM of the NiH_3_L1 complex. In this case, the increase in mitochondrial activity was even greater than in the negative control. This suggests that the beneficial properties of L1 are complexed with Ni(II) ions for the potential treatment of infected wounds. At the same time, L1 and L3 at a concentration of 100 µM, and L2 at concentrations of 10 and 50 µM significantly enhanced the mitochondrial activity compared to that of the positive control. Complexation of these compounds with copper ions increased the mitochondrial activity of NHDF as compared to that of the positive control, but to a lesser extent than complexing these compounds with Ni(II) ions.

An increase in oxygen-free radical species (ROS) is ubiquitous in bacterial infections. At the same time, chronic oxidative stress is undesirable in wound healing. Therefore, we examined how the tested compounds affected the ROS level after pre-incubation with LPS (Figure 11B). It was found that in the positive control, a statistically significant increase of approximately 138% in ROS took place. At the same time, the use of the test compounds and their complexes at all tested concentrations resulted in a reduction of the ROS level in relation to the positive control (statistically significant). It should be noted that both the copper and nickel complexes lowered the ROS level to the negative control level. On the other hand, without complexation, the compounds reduced ROS but only to a level of about 118% (except for compound L3 at 100 µM, which lowered ROS to the negative control level).

#### 2.4.2. Anti-Inflammatory Activity

**Figure 12:**
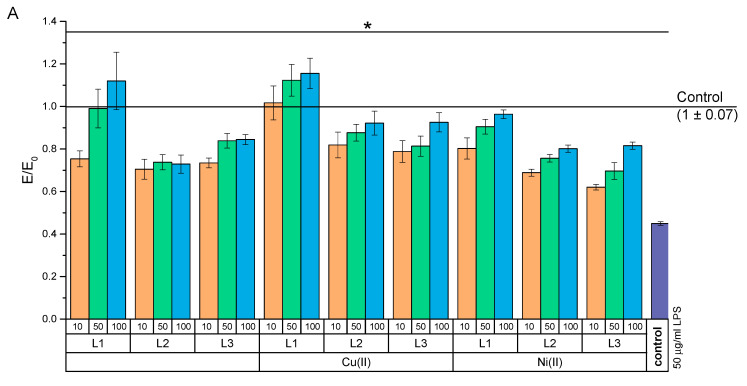

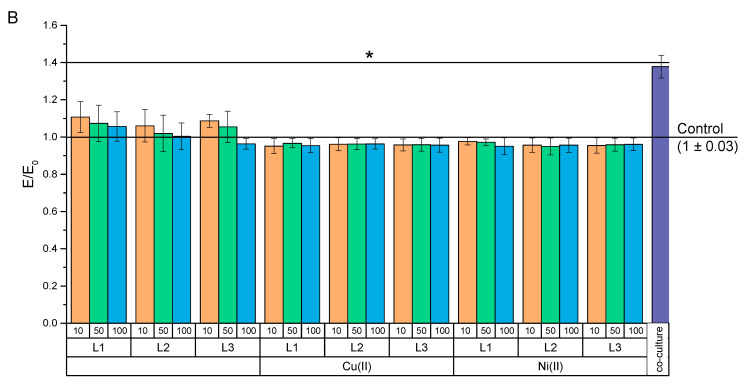
Effect of test compounds on NHDF cells co-cultured with THP-1 cells: (**A**) MTT test and (**B**) DCF-DA test. Control—cell culture incubated without co-culture with THP-1 and test substances, and * *p* < 0.05—significant difference as compared to that of the control preincubated with co-culture containing THP-1.

In addition to bacterial infections, chronic inflammation is a serious problem in wound healing. Therefore, the effect of test compounds on the inflammation induced by co-culturing NHDF and THP-1 (PMA-activated) cells was investigated (Figure 12). The tested compounds and their complexes significantly increased the mitochondrial activity of cells preincubated in co-culture with THP-1 over the entire range of the concentrations (Figure 11A). At the same time, the largest increase in mitochondrial activity occurred at a concentration of 100 µM for L1 and over the entire concentration range of this drug after complexing it with copper ions. Moreover, over the concentration range of 50–100 µM of the CuH_3_L1 complexes, an increase in mitochondrial activity took place as compared to that of the negative culture. In addition, the tested compounds and their complexes showed a statistically significant reduction of the ROS level as compared to that of the positive control (Figure 12B).

## 3. Materials and Methods

### 3.1. Synthesis and Purification of the Compounds

Peptides L1 and L2 were synthesized manually by the solid-phase Fmoc/tBu method. Amino acids used in this study were Fmoc-L-Ala-OH, Fmoc-L-Arg(Pbf)-OH, Fmoc-Gly-OH, Fmoc-L-His(Trt)-OH, Fmoc-L-Lys(Boc)-OH, Fmoc-L-Pro-OH, (Orpegen Peptide Chemicals GmbH, Heidelberg, Germany), Fmoc-L-Dab(Boc)-OH (Carbolution Chemicals GmbH, St. Ingbert, Germany), and Fmoc-L-Orn(Boc)-OH (Ambeed, Inc., Arlington Heights, IL, USA). Peptide L1 was synthesized on 2-chlorotritile chloride resin (2-CTC, loading 1.6 mmol/g, CBL Patras, Patra, Greece) and peptide L2 on Wang resin (Sunresin, loading 0.71 mmol/g, Xi’an Hi-tech Industrial Development Zone, Shaanxi, China) as a solid support. Dbt (1,4-diaminobutane, 1 eq, Merck, Darmstadt, Germany) was attached to 2-CTC resin through incubation with DIPEA (2 eq, *N,N*-diisopropylethylamine, Iris Biotech GmbH, Marktredwitz, Germany) using threefold excess based on the resin for 2 h at RT. DCM (dichloromethane, Chempur, Piekary Slaskie, Poland) was used as a solvent. The unreacted resin was terminated with methanol (1 mL per gram of the resin, Chempur, Piekary Slaskie, Poland). Fmoc-L-Arg(Pbf)-OH (1 eq) was conjugated with the Wang resin linker. The reaction was carried out with arginine derivatives, specifically DMAP (4-(dimethylamino)pyridine, 0.1 eq, Merck, Darmstadt, Germany) and DIC (*N,N′*-diisopropylcarbodiimide, Carbolution Chemicals GmbH, St. Ingbert, Germany) dissolved in DCM. Unreacted Wang resin linker was blocked with acetic anhydride (Chempur, Piekary Slaskie, Poland) and DIPEA (1:1, 1 mmol/g of resin) for 30 min. Deprotection of the Fmoc group was accomplished with a 20% piperidine (Iris Biotech GmbH, Marktredwitz, Germany) solution in N,N-dimethylformamide (DMF; POCH, Avantor, Gliwice, Poland) for 15 min. Acylation was performed with an equimolar mixture of *N,N’*-diisopropylcarbodiimide (DIC), OxymaPure, and Fmoc-AA-OH dissolved in DCM:DMF (1:1, *v/v*) in four-fold excess based on the resin for 1.5 h (OxymaPure; Carbolution Chemicals GmbH, St. Ingbert, Germany). After deprotection and coupling reactions, the resin was rinsed with DMF and DCM, and, subsequently, the chloranil test was carried out. Peptides L1 and L2 were cleaved from the resin using a mixture of TFA (trifluoroacetic acid, Apollo Scientific, Denton, UK), TIS (triisopropylsilane, Iris Biotech GmbH, Marktredwitz, Germany), and demineralized water (95:2.5:2.5, *v/v*). This stage was accomplished at RT within 1.5 h with agitation. Crude peptides were precipitated with cooled diethyl ether (POCH, Avantor, Gliwice, Poland) and centrifuged. Precipitates were dissolved in water and lyophilized. Purifications were performed by RP-HPLC (C18 column, gradient-grade water and acetonitrile with 0.1% TFA, *v/v*). Eluents were purchased from POCH (Avantor, Gliwice, Poland). Identity was confirmed with ESI-MS (Table S1). Pure fractions were collected (>95%, by RP-HPLC) and lyophilized (Figures S2 and S3).

The L3 peptide was commercially ordered in the STI company, Poznań, Poland. The purity of the pseudopeptide compounds was higher than 95%, as confirmed by the potentiometric Gran method. Identity was also confirmed with ESI-MS (Table S1).

FT-IR were used to characterize all analyzed ligands (Figures S4–S6).

### 3.2. Potentiometric Titration

The acidic solvent for the potentiometric measurements was prepared by dilution of a 37% HCl (Sigma Aldrich, Poznan, Poland) to obtain 3.16 × 10^−3^ M solution and an appropriate amount of solid KCl (Sigma Aldrich, Poznan, Poland) was added to obtain an ionic strength of 0.1 M in the final solution. The 0.1 M standard solution of KOH (Merck, Darmstadt, Germany) was used as a titrant. The precise titer was determined by multiple titrations of the potassium hydrogen phthalate (Sigma Aldrich, Poznan, Poland) solution and averaging the results. The stock solutions of metal ions were prepared by dissolving solid CuCl_2_·2H_2_O (Merck, Darmstadt, Germany) and NiCl_2_·6H_2_O (Merck, Darmstadt, Germany) in the acid. The concentrations of metal ions were determined spectrophotometrically. All reagents were of analytical grade.

The potentiometric titrations were performed on a Titrando 907 by Metrohm (Metrohm AG, Herisau, Switzerland) equipped with a combined Biotrode^®^ electrode (Metrohm AG, Herisau, Switzerland). The standard calibration procedure was carried out before measurements [53]. The electrode parameters (E^0^ and S_L_) were calculated using the Nernst Equation (1):

(1) $$E^{0}=E+2.303\frac{RT}{F}log\left[H^{+}\right]S_{L}$$

Solutions of ligands (L1, L2, and L3) were prepared by dissolving them in 3.16 × 10^−3^ M HCl (pH 2.5) with an ion strength I = 0.1 M. The volumes of the solutions were 1.5–2.0 mL and the ligand concentration range was 10.0–7.0 × 10^−4^ M, as determined by the Gran method [54,55]. Titrations were carried out in a thermostat vessel at 25 °C under Ar atmosphere (5.0). Metal ion solutions were prepared by adding CuCl_2_ and NiCl_2_ stock solutions. Metal-to-ligand molar ratios in the final solutions were equal. A CO_2_-free standard 0.1 M KOH titrant was used. The titration curves were provided over the pH range of 2.5–11.5 for all measurements. This enabled the recording of about 160–200 points per curve.

The fitting procedure to experimental curves was calculated using the HYPERQUAD [56] computer program with the aid of a non-linear least-squares algorithm. The potentiometric data allowed for the establishment of protonation constants *(β_i_* = [H_i_L]/[H^+^]^i^[L]) of the ligands in accordance with equilibrium reaction (2):

(2) $$iH+L↔H_{i}L$$

The formation of the constants of the complexes was calculated by following equilibrium reaction (3 and 4):

(3) $$pM+qH+rL↔M_{p}H_{q}L_{r}$$

(4) $$\beta_{pqr}=\frac{\left[M_{p}H_{q}L_{r}\right]}{[M{]}^{p}[H{]}^{q}{\left[L\right]}^{r}}$$

where M = Cu(II) or Ni(II), and L = non-protonated ligands L1, L2, and L3 whose charges were omitted for simplicity. The standard deviations were computed for all constants using the same program and they indicated only random errors. The species distribution diagrams were prepared in the HYSS program [57].

### 3.3. Spectroscopic Measurements (UV-Vis and CD)

Sample solutions with metal ions were prepared similarly to those for potentiometric titration. All spectroscopic measurements were carried out at 25 °C. The pH of the samples was determined by adding a small quantity of the concentrated KOH solutions and was controlled for by the Mettler Toledo pH-meter Five Easy F20 (Mettler Toledo, Columbus, OH, USA) equipped with the combined electrode InLab^®^Micro (Mettler Toledo, Columbus, OH, USA). The absorption spectra of the complexes were recorded on a Jasco spectrophotometer (Jasco, Tokyo, Japan) over the range 350–900 nm at 0.1 nm intervals. All spectra were collected in quartz cells with a 1 cm path length. For each spectrum, the molar extinction coefficients (ε [dm^3^·mol^−1^cm^−1^]) at absorption bands based on the Lambert–Beer law were calculated.

The circular dichroism (CD) spectra were recorded on a JASCO J 1500 magnetic circular dichroism spectrometer (Jasco, Tokyo Japan). The optical length of the quartz cuvettes was 1 cm and the spectral range was 230–800 nm with 0.1 nm resolution. The measurements were carried out in an inert gas atmosphere (N_2_). The CD spectral data were recalculated to the molar circular dichroism (Δε [Μ^−1^cm^−1^]) by Jasco analysis software using Equation (5):

(5) $$\Delta \varepsilon =\frac{\theta_{exp}}{3298\times c\times l\times 10}$$

in which *ϴ_exp_* is the experimental ellipticity, *c* is the molar concentration of complex, and *l* is the optical path length of the cuvettes.

The results obtained from spectroscopic methods, together with potentiometric methods, allowed for defining the numbers and types of donors around the metal ion.

### 3.4. Mass Spectrometry Measurements

High-resolution mass spectra were acquired on a compact™ Bruker Daltonics spectrometer (GmbH Bruker, Mannheim, Germany) with a standard ESI source. The instrument was calibrated before each measuring series by the Tunemix™ mixture. Data were collected in the positive ion mode. The samples were injected at a flow rate of 3 μL/min under the drying gas (N_2_) at 200 °C, while the collision voltage was 4.0 kV at a flow rate of 4.0 L/min. The samples were prepared in ultrapure water by dissolving powdered ligands and adding appropriate volumes of CuCl_2_ and NiCl_2_ stock solutions. The final ligand concentration was 0.5–1 × 10^−4^ M.

Mass spectrometry was used to check the purity of the analyzed compounds and to confirm the formation of complex forms with a given stoichiometry.

### 3.5. Biological Evaluation

#### 3.5.1. Cell Line and Culture Media

Normal human dermal fibroblast cells (NHDF-Ad—Lonza CC-2511; Basel, Switzerland) and the human monocyte cell line (THP-1—ECACC 88081201—obtained from Merck Millipore; Darmstadt, Germany) were used in the study. Cells were grown at 37 °C in 5% CO_2_ and 95% humidity with both morphology and confluence assessed twice a week. If confluence was above 70%, cells were passaged (TrypLE solution was used for NHDF, which are adherent cells) or the medium was replaced with fresh one. THP-1 was grown in suspension. Then, the cells were collected in tubes each time and centrifuged at 1000× *g* for 5 min. Then, the supernatant was removed and a fresh medium was added, after which the cells were resuspended and transferred to culture flasks (all or reduced amounts when confluence was above 70%). For NHDF cells, DMEM without red phenol medium was used, while THP-1 cells were incubated in RPMI-1960. Both media were supplemented with 10% Fetal Bovine Serum (FBS) and 2 mM of both L-glutamine and antibiotics (gentamicin and streptomycin).

#### 3.5.2. Cytotoxicity Evaluation

Assessment of the cytotoxicity of new compounds and their complexes with metal ions (Cu(II) and Ni(II)) was performed using the MTT assay. This test has been recommended for the evaluation of the cytotoxicity of new compounds and biomaterials. The evaluation was carried out according to ISO10993-5, part C. NHDF was used in this test because these fibroblasts were of human origin, thus they provide a better model than those of L929 or Balb3T3 (mouse fibroblasts). In the first step, cells were seeded at 10,000 cells/well and incubated overnight at 37 °C in 5% CO_2_ and 95% humidity to allow cells to adhere to the well surface. The next day, the concentration of the test compounds and their complexes was prepared in the medium for NHDF cells, but with a reduced concentration of FBS to 5%. Concentrations of 10, 50, and 100 µM were tested. After 30 min of complexation of compounds, the supernatant from the culture plates was removed and the test substances were added. Cells with compounds were incubated for 24 h. After this time, an MTT solution was prepared. MTT powder was dissolved in MEM without FBS and phenol red was reduced to a 1 mg/mL concentration. Cell morphology was assessed microscopically on a 5-point scale. The supernatant was then removed and the MTT solution was added for 2 h at 37 °C in 5% CO_2_ and 95% humidity. The supernatant was then carefully removed and the purple crystals were dissolved in 100 µL of isopropanol for 30 min with shaking. Finally, the absorbance at 570 nm was measured with a Multiskan GO microplate reader (Thermo Fisher Scientific; Waltham, MA, USA).

#### 3.5.3. Experimental Design

MTT and DCF-DA assays were performed to assess mitochondrial activity and oxygen-free radical levels, respectively. The tests were carried out in two models. The first model was used to mimic difficult-to-heal wounds with enhanced inflammation. This model was designed using the co-cultivation of two cell lines. THP-1 cells, after seeding at a density of 40,000 cells per well, were treated with 5 ng/mL of PMA (this factor activates THP-1 cells to secrete proinflammatory cytokines). The medium was changed every 48 h for 5 days, collecting the supernatant and storing it at 4–8 °C. On the fourth day of the culture of THP-1 in the presence of PMA, new plates were seeded with fibroblast cells. After NHDF adhered to the surface of the wells overnight, the medium was removed and the combined 5-day culture supernatant THP-1 (rich in proinflammatory cytokines) was added to the culture. This supernatant was combined, mixed, and left at 37 °C for 1 h before being used in NHDF culture. Then, the co-culture was incubated for 24 h. Another model enabled the assessment attenuation of LPS-induced cytotoxicity. An NHDF cell culture was treated with 50 µg/mL of LPS or with a supernatant rich in proinflammatory cytokines from THP-1 cell cultures for 24 h. After this time, the supernatant was removed, regardless of whether it was a co-culture of LPS or THP-1, and test compounds and their complexes were added. The tests were carried out over a concentration range of 10–100 µM for the test compounds. These concentrations were prepared 30 min before the end of the 24 h incubation with harmful agents (LPS or co-culture). Then, the culture was incubated again for another 24 h. After this time, the MTT and DCF-DA assays were performed. The first assay was carried out in accordance with the procedure described in Section 3.5.2. In assessing the level of free-oxygen radicals, the supernatant was removed and a freshly prepared solution of 25 µM of DCF-DA in PBS was added for 1 h. After this time, fluorescence readings were performed at a length ex 535 nm and 498 nm using a Multiskan GO microplate reader (Thermo Fisher Scientific; Waltham, MA, USA).

#### 3.5.4. Statistical Analysis

All bioassays were performed in 3 independent replicates, each with 5 replicates per group. The results are presented as the ratio E/E_0_, where E is the result from a given compound at a given concentration, and E_0_ are those results from a culture without the tested compounds and no harmful factors (negative control). Positive controls were additionally used in studies evaluating antibacterial and anti-inflammatory effects, particularly regarding cultures of cells treated with LPS only or with a supernatant rich in proinflammatory cytokines from THP-1 cell cultures. Differences between concentrations and compounds were assessed using one-way ANOVA and Tukey’s post-hoc tests. The point of significance was *p* < 0.05. The statistical evaluation was performed in the Statistica v13.3 program (StatSoft, Krakow, Poland).

## 4. Conclusions

The peptides studied revealed to be quite convenient chelators for divalent metal ions such as Cu(II) and Ni(II). The results showed that three ligands form thermodynamically stable complexes with Cu(II) and Ni(II) ions above pH 4.0 and 6.0, respectively. At the crucial pH region (including the physiological 7.4 point), the copper complexes with L1 and L3 ligands form mostly CuH_5_L1 and CuH_3_L3 species, where the metal ions are coordinated by four nitrogen atoms (4N) in a square–planar orientation. The coordination process begins at the *N*-terminal peptide fragment, with high stability of the complexes, and can probably be a consequence of the presence of at least two adjacent Dab residues in the peptide sequence, whose amino side chain groups are engaged in complexation. In contrast, the L2 compound, with one Dab entity only in the structure, creates at neutral pH CuH_3_L2 species with a three-nitrogen (3N) donor set. In nickel complexes, NiH_5_L1, NiH_3_L2, and NiH_3_L3 forms dominate at physiological pH. The L1 and L3 peptides are characterized by identical coordination modes with four nitrogen atoms in the coordination sphere of the metal ion. In NiH_3_L2, three nitrogen donors from the ligand molecule interact with the metal ion. Nonetheless, only L2 offers third amide nitrogen to form NiH_-1_L2 with the {NH_2_, 3 × N^−^} coordination mode under a highly basic solution. Despite the presence of His residue in L1 and L2 sequences, which is a strong coordinating amino acid, the imidazole nitrogen atom is not involved in metal ions binding. Moreover, the order in the coordination efficiency for both Cu(II) and Ni(II) is as follows: L1 > L2 > L3.

In addition, we also performed biological evaluations of all the ligands. Both the tested compounds and their complexes showed no cytotoxicity. Results of the attenuation of the LPS-induced cytotoxicity test showed that L1 complexed with Ni(II) ions had optimal characteristics and could be useful for the treatment of bacterial infections. Additionally, encouraging results were obtained for L1, L2, and L3 complexes with Cu(II) ions. Moreover, all the tested complexes reduced the level of oxygen-free radical species (ROS) over the physiological range of pH. Anti-inflammatory activity tests showed that the analyzed compounds and their complexes enhanced the mitochondrial activity of NHDF cells preincubated in co-culture with THP-1.

In summary, quite satisfactory results were obtained for the three tested peptides and their complexes in regard to their activity. They are promising compounds for further studies and use in medicine.

## Figures and Tables

**Table 1:** The overall protonation constants (log*β_i_*) and stepwise constants (log*K_i_*) were calculated for L1, L2, and L3 ligands derived from the potentiometric curves.

	L1		L2		L3	
Form	log*β_i_*	log*K_i_*	log*β_i_*	log*K_i_*	log*β_i_*	log*K_i_*
H_10_L	91.45 ± 0.05	5.86				
H_9_L	85.59 ± 0.05	6.92				
H_8_L	78.67 ± 0.05	8.45	67.25 ± 0.03	2.50	69.10 ± 0.02	2.64
H_7_L	70.22 ± 0.05	8.94	64.75 ± 0.03	5.96	66.47 ± 0.02	6.77
H_6_L	61.29 ± 0.05	9.39	58.79 ± 0.03	7.18	59.70 ± 0.02	8.50
H_5_L	51.90 ± 0.07	9.64	51.60 ± 0.03	9.00	51.20 ± 0.02	9.10
H_4_L	42.26 ± 0.04	10.08	42.60 ± 0.03	9.73	42.09 ± 0.02	9.73
H_3_L	32.18 ± 0.10	10.21	32.87 ± 0.03	10.22	32.36 ± 0.03	10.14
H_2_L	21.97 ± 0.02	10.82	22.65 ± 0.02	11.03	22.22 ± 0.01	10.91
HL	11.15 ± 0.11	11.15	11.62 ± 0.04	11.62	11.31 ± 0.03	11.31

log*K_i_* = log*β_i_*−log*β_i−1_*_._

**Table 2:** The calculated stability constants (log*β_pqr_*) and stepwise constants (log*K_pqr_*) were obtained from the potentiometric titration for equimolar systems: L:Cu(II) = 1:1.

	L1		L2		L3	
Complex Form	log*β_pqr_*	log*K_pqr_*	log*β_pqr_*	log*K_pqr_*	log*β_pqr_*	log*K_pqr_*
CuH_7_L	80.58 ± 0.01	4.54				
CuH_6_L	76.04 ± 0.01	6.46				
CuH_5_L	69.59 ± 0.01	9.08				
CuH_4_L	60.50 ± 0.01	9.51	53.25 ± 0.03	5.28	55.24 ± 0.06	4.84
CuH_3_L	50.99 ± 0.01	9.83	47.97 ± 0.03	9.36	50.40 ± 0.03	9.39
CuH_2_L	41.16 ± 0.01	10.12	38.60 ± 0.06	9.79	41.01 ± 0.08	9.82
CuHL	31.04 ± 0.01	10.46	28.81 ± 0.05	10.37	31.19 ± 0.07	10.33
CuL	20.58 ± 0.02	10.78	18.44 ± 0.08	10.96	20.86 ± 0.08	10.88
CuH_−1_L	9.80 ± 0.01	11.38	7.48 ± 0.09	11.13	9.98 ± 0.07	
CuH_−2_L	−1.58 ± 0.01		−3.65 ± 0.07			

logK_pgr_ = logβ_pqr_−logβ_p(q−1)r._

**Table 3:** The calculated stability constants (log*β_pqr_*) and stepwise constants (log*K_pqr_*) were obtained from potentiometric titration for equimolar systems: L:Ni(II) = 1:1.

	L1		L2		L3	
Complex Form	log*β_pqr_*	log*K_pqr_*	log*β_pqr_*	log*K_pqr_*	log*β_pqr_*	log*K_pqr_*
NiH_6_L	68.79 ± 0.02	7.26				
NiH_5_L	61.53 ± 0.03	18.21				
NiH_4_L			48.21 ± 0.05	6.69		
NiH_3_L	43.32 ± 0.04	20.09	41.52 ± 0.02	18.94	42.38 ± 0.02	9.26
NiH_2_L					33.13 ± 0.04	9.65
NiHL	23.23 ± 0.05	10.25	22.58 ± 0.02	20.88	23.47 ± 0.05	10.38
NiL	12.98 ± 0.05	21.75			13.09 ± 0.05	10.47
NiH_-1_L			1.70 ± 0.03	11.05	2.63 ± 0.03	
NiH_-2_L	−8.77 ± 0.05		−9.35 ± 0.05	11.62		
NiH_-3_L			−20.97 ± 0.08			

logK_pgr_ = logβ_pqr_−logβ_p(q−1)r_.

## Supplementary Materials

The following are available online at https://www.mdpi.com/article/10.3390/ijms222112028/s1.
