# Supplementary material for: Cationic Peptides and Their Cu(II) and Ni(II) Complexes: Coordination and Biological Characteristics
Source: Int J Mol Sci. 2021 Nov 6;22(21):12028. doi: 10.3390/ijms222112028 (PMC8584440; doi:10.3390/ijms222112028)
Supplement: Supplementary file 1 [file ijms-22-12028-s001.zip › ijms-1429978-supplementary.pdf]

Supplementary Materials

Cationic Peptides and their Cu(II) and Ni(II) Complexes: Coordination and Biological Characteristics

Aleksandra Kotynia [1,*], Benita Wiatrak [2], Wojciech Kamysz [3], Damian Neubauer [3], Paulina Jawien [2,4] and Aleksandra Marciniak [1,*]

Table of Contents

Table S1. MS data of the peptides: L1, L2, L3. ……………………………………………………………...S3

Figure S2. Analytical HPLC data of peptide L1. ……………………………………………..……………S3

Figure S3. Analytical HPLC data of peptide L2. ……………………………………………..….………S4

Figure S4. FT-IR spectra for peptide L1. …………………………………………………….……………..S4

Figure S5. FT-IR spectra for peptide L2. …………………………………………………….……………..S5

Figure S6. FT-IR spectra for peptide L3. …………………………………………………….……...……S5

Table S7. The UV-Vis and CD spectroscopic data for equimolar L1:Cu(II) system. ……….……………S6

Table S8. The UV-Vis and CD spectroscopic data for the equimolar L2:Cu(II) system. ………………...S7

Table S9. The UV-Vis and CD spectroscopic data parameters for equimolar L3:Cu(II) system. ……….S8

Table S10. The UV-Vis and CD spectroscopic data for equimolar L1:Ni(II) system. ……………………S9

Table S11. The UV-Vis and CD spectroscopic data for equimolar L2:Ni(II) system. …………………...S10

Table S12. The UV-Vis and CD spectroscopic data parameters for equimolar L3:Ni(II) system. …….S11

Table S1. MS data of the peptides L1 and L2, L3.

Peptide	Structure	z	m/z calc.	m/z exp.
L1	Lys-Dab-Dab-Gly-Orn-Pro-His-Lys-Arg-Lys-Dbt	1<br>2	1234.83<br>617.92	1235.10<br>618.42
L2	Lys-Dab-Ala-Gly-Orn-Pro-His-Lys-Arg	1<br>2<br>3<br>4	1007.62<br>504.31<br>336.55<br>252.66	1007.74<br>504.58<br>337.05<br>253.31
L3	Lys-Dab-Dab-Gly-Orn-Pro-Phe(2F)-Lys-Arg	1<br>2<br>3	1063.64<br>532.82<br>355.55	1064.66<br>532.83<br>355.56

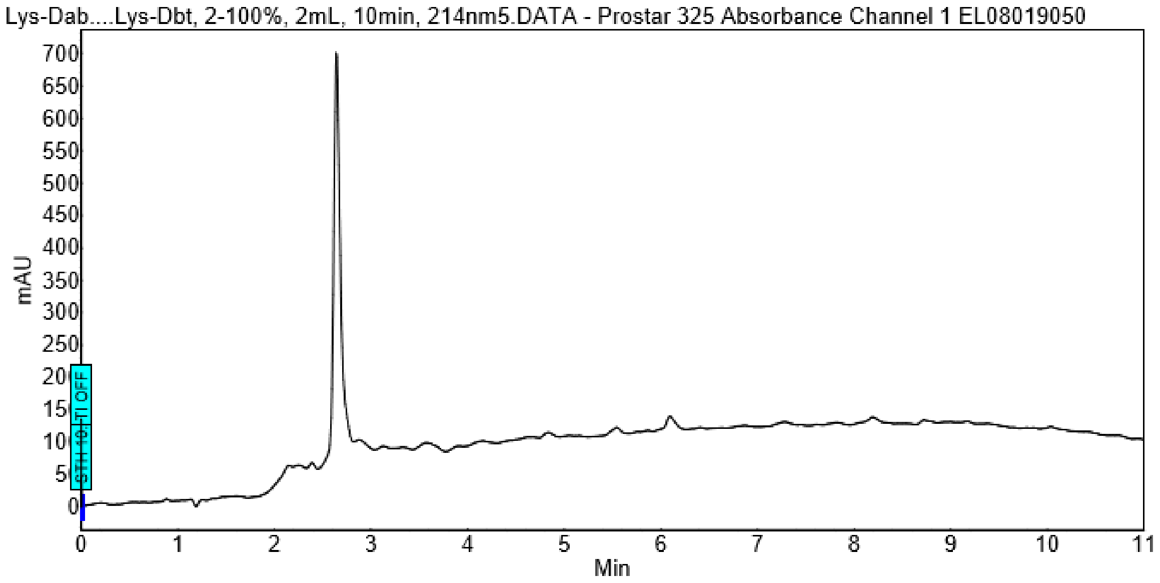

Figure S2. Analytical HPLC data of peptide L1.

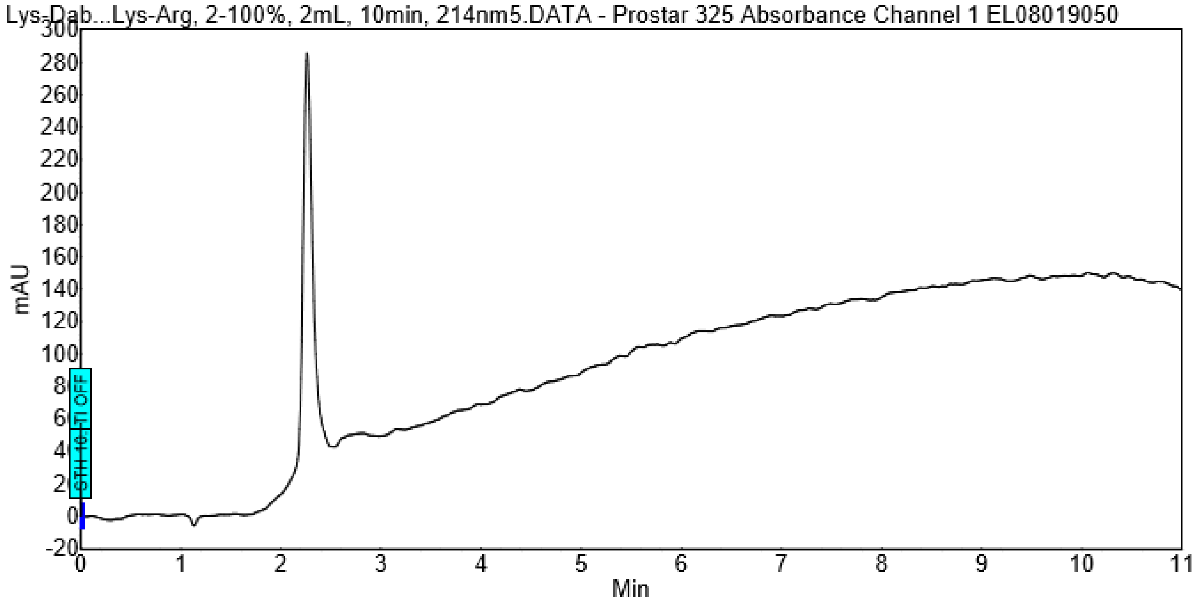

Figure S3. Analytical HPLC data of peptide L2.

The purity of the peptides was determined on a Varian ProStar HPLC system controlled by a Galaxie Chromatography Data System. Analyses were carried out on a Phenomenex Luna C18(2) column (4.6 × 150 mm, 110 Å pore size, 5 µm particle size). The solvent systems used were: 0.1% aqueous TFA (A) and 0.1% TFA in acetonitrile (ACN) (B). The peptides were eluted with a linear gradient 2–100% B in A over 10 min at 25 ± 0.1 °C. UV detection at 214 nm was used and the mobile phase flow rate was 2.0 mL/min.

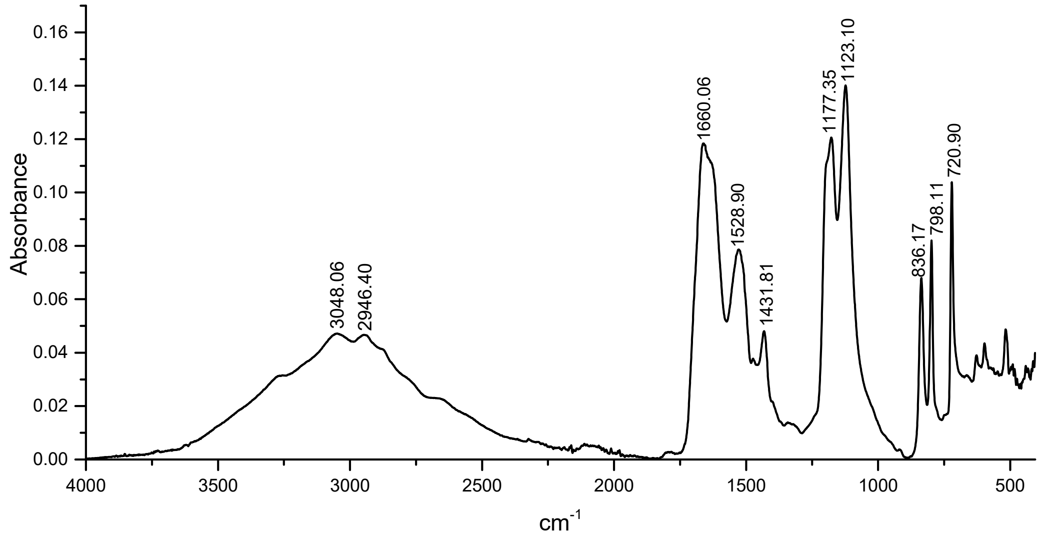

Figure S4. FT-IR spectra for peptide L1.

FT-IR (selected lines, γ_{max}, cm^{-1}): 3048.06 (Amide B: ν(NH)), 2946.40 (Amide B: ν(NH) and νCH), 1660.06 (Amide I: ν(C=O)), 1528 ($\nu(NH_2)$), 1431.81 ($\delta(O{=}C{-}CH_2)$), 1177.35 (ν(CN), 1123.10, 836.17, 798.11, 720.90 (fingerprint region).

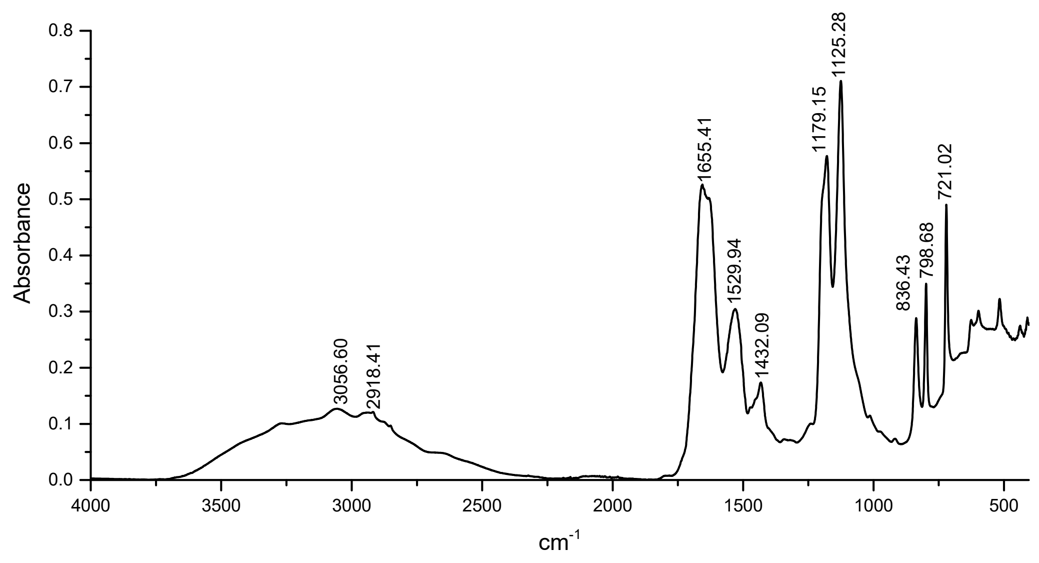

Figure S5. FT-IR spectra for peptide L2.

FT-IR (selected lines, γ_{max}, cm^{-1}): 3056.60 (Amide B: ν(NH)), 2918.41 (Amide B: ν(NH) and νCH), 1655.41 (Amide I: ν(C=O)), 1529.94 (ν(NH_2)), 1432.09 (δ(O=C–CH_2)), 1179.15 (ν(CN), 1125.28, 836.43, 798.68, 721.02 (fingerprint region).

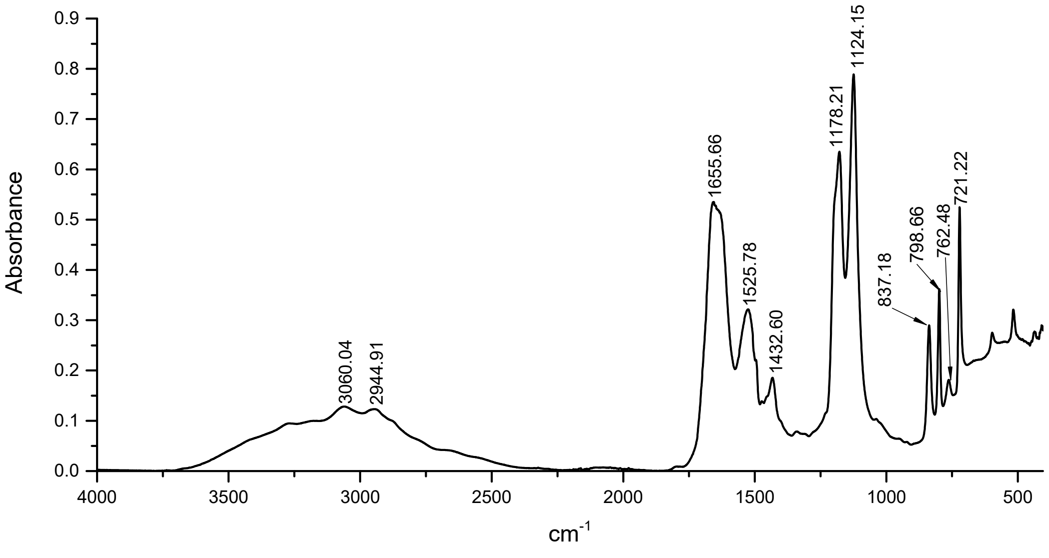

Figure S6. FT-IR spectra for peptide L3.

FT-IR (selected lines, γ_{max}, cm^{-1}): 3060.04 (Amide B: ν(NH)), 2944.91 (Amide B: ν(NH) and νCH), 1655.66 (Amide I: ν(C=O)), 1525.78 (ν(NH_2)), 1432.60 (δ(O=C–CH_2)), 1178.21 (ν(CN), 1124.15, 837.18, 798.66, 762.68 (νAr-F), 721.22 (fingerprint region).

Infrared spectra were recorded on Nicolet iS50 FTIR (Thermo Scientific) equipped with deutered triglicyne sulphate (DTGS) detector and KBr beam splitter. The spectral data were recorded within range of 4000 to 400 cm-1 with resolution 4cm-1 and 64 scans were averaging. The spectra were obtained at room temperature, the powder sample of each peptides (L1, L2, L3) was applied on the crystal window and measured by using Attenuated Total Reflectance (ATR) method. The data analysis was proceeded by Omnic 9.3.30 (Thermo Fisher Scientific Inc.) software.

Table S7. The UV-Vis and CD spectroscopic data for equimolar L1:Cu(II) system.

System	pH	UV-Vis		CD	
		λ [nm]	ε [$M^{-1}cm^{-1}$]	λ [nm]	Δε [$M^{-1}cm^{-1}$]
L1:Cu(II)	4.55	570[a]	7.9	238[b]	−0.68
	5.03	542[a]	57.7	553[a]	−0.08
				480[a]	0.07
				296[b]	0.60
				250[b]	−1.91
	5.51	543[a]	68.3	553[a]	−0.13
				476[a]	0.10
				297[b]	0.77
				252[b]	−2.05
	6.08	533[a]	73.17	553[a]	−0.19
				479[a]	0.13
				300[b]	0.92
				256[b]	−1.94
	6.54	529[a]	78.3	553[a]	−0.22
				479[a]	0.14
				301[b]	1.10
				259[c]	−2.21
	7.13	553[a]	78.3	553[a]	−0.28
				479[a]	0.19
				303[b]	1.36
				264[c]	−2.73
	7.54	525[a]	78.3	553[a]	−0.30
				480[a]	0.19
				303[b]	1.44
				264[c]	−2.93
	8.07–10.51	524[a]	88.3	553[a]	−0.30
				480[a]	0.19
				304[b]	1.50
				266[c]	−3.09
	11.04	520[a]	91.6	553[a]	−0.30
				480[a]	0.19
				304[b]	1.50
				266[c]	−2.47

[a] d-d transition [b] NH_2 → Cu(II) CT [c] N^- → Cu(II) CT.

Table S8. The UV-Vis and CD spectroscopic data for the equimolar L2:Cu(II) system.

System	pH	UV-Vis		CD	
		λ [nm]	ε [$M^{-1}cm^{-1}$]	λ [nm]	Δε [$M^{-1}cm^{-1}$]
L2:Cu(II)	5.02	605[a]	39	288[b]	0.16
				245[c]	−1.27
	5.57	577[a]	70	277[b]	0.60
				249[c]	−2.11
	6.18	560[a]	70	277[b]	0.79
				249[c]	−2.38
	6.55–8.00	557[a]	74.5–82.5	277[b]	1.10–1.18
				249[c]	−2.03–(−2.28)
	8.57	-		570[a]	−0.14
			-	277[b]	0.94
				249[c]	−2.43
	9.00	552[a]	80.3	570[a]	−0.13
				277[b]	1.11
				249[c]	−1.90
				570[a]	−0.13
	9.52	-	-	277[b]	0.94
				249[c]	−1.73
		544[a]	87.8	557[a]	−0.18
	10.08			277[b]	0.94
				249[c]	−1.14
		532[a]	93.6	538[a]	−0.36
	10.52			280[b]	0.68
				252[c]	−0.54
		518[a]	113.3	529[a]	−0.77
	11.00			300[b]	0.30
				266[c]	−0.31
		509[a]	145.5	523[a]	−1.38
	11.56			303[b]	0.55
				268[c]	−1.24

[a] d-d transition [b] $NH_2 \rightarrow$ Cu(II) CT [c] $N^- \rightarrow$ Cu(II) CT.

Table S9. The UV-Vis and CD spectroscopic data parameters for equimolar L3:Cu(II) system.

System	pH	UV-Vis		CD	
		λ [nm]	ε [$M^{-1}cm^{-1}$]	λ [nm]	Δε [$M^{-1}cm^{-1}$]
L3:Cu(II)	5.0	542[a]	43.6	300[b]	0.55
	5.49	530[a]	86.9	558[a]	−2.04
				475[a]	0.10
				301[b]	1.23
				265[c]	−2.17
	6.19	525[a]	101.6	558[a]	−0.32
				475[a]	0.14
				302[b]	1.49
				265[c]	−2.80
	6.63–7.45	523[a]	104.6–106.6	558[a]	−0.35
				475[a]	0.18
				304[b]	1.59
				265[c]	−3.04
	8.04-9.50	525[a]	106.6	558[a]	−0.37
				475[a]	0.18
				304[b]	1.61
				265[c]	−2.82
	10.0-11.0	520[a]	111.5	553[a]	−0.39
				475[a]	0.197
				305[b]	1.67
				266[c]	−2.43

[a] d-d transition [b] NH_2 → Cu(II) CT [c] N^- → Cu(II) CT.

Table S10. The UV-Vis and CD spectroscopic data for equimolar L1:Ni(II) system.

System	pH	UV-Vis		CD	
		λ [nm]	ε [$M^{-1}cm^{-1}$]	λ [nm]	Δε [$M^{-1}cm^{-1}$]
L1:Ni(II)	7.02	429[a]	62.5	477[a]	−0.68
				417[a]	0.33
				258[c]	1.78
	7.50	429[a]	94.9	477	−1.16
				413	0.47
				258[c]	2.70
	8.06	429[a]	104.9	475	−1.35
				413	0.51
				258[c]	2.98
	8.58–10.03	427[a]	107.8	475	1.41
				413	054
				258	3.04
	10.52	426[a]	117.8	475	1.41
				413	0.40
				258	3.25
	11.03	425[a]	122.5	472	−1.54
				411	0.16
				258[c]	3.84

[a] d-d transition [b] $NH_2 \rightarrow Ni(II)$ CT [c] $N^- \rightarrow Ni(II)$ CT.

Table S11. The UV-Vis and CD spectroscopic data for equimolar L2:Ni(II) system.

system	pH	UV-Vis		CD	
		λ [nm]	ε [$M^{-1}cm^{-1}$]	λ [nm]	Δε [$M^{-1}cm^{-1}$]
L2:Ni(II)	7.0	450[a]	35.7	497[a]	−0.23
				440[a]	0.23
				260[c]	0.50
				239[b]	−2.80
	7.59	448[a]	72.0	497[a]	−0.30
				440[a]	0.40
				260[c]	−0.33
				239[b]	−3.0
	8.07	448[a]	82.6	497[a]	−0.33
				435[a]	0.36
				260[c]	1.36
				239[b]	−2.56
	8.55	448[a]	88.1	491[a]	−0.47
				435[a]	0.31
				260[c]	1.63
				239[b]	−1.41
	9.17	444[a]	94.5	485[a]	−0.62
				435[a]	0.25
				260[c]	1.86
	9.54	435[a]	102.5	486[a]	−0.63
				435[a]	0.09
				260	2.40
	10.07	416[a]	140.8	482[a]	−1.0
		448[sh]		260[c]	3.48
	10.54	408[a]	210.6	468[a]	−2.07
				268[c]	4.20
	11.04–11.50	408[a]	227.0	460[a]	−2.80
				271[c]	3.63

[a] d-d transition [b] $NH_2 \rightarrow Ni(II)$ CT [c] $N^- \rightarrow Ni(II)$ CT [sh] shoulder.

Table S12. The UV-Vis and CD spectroscopic data parameters for equimolar L3:Ni(II) system.

System	pH	UV-Vis		CD	
		λ [nm]	ε [$M^{-1}cm^{-1}$]	λ [nm]	Δε [$M^{-1}cm^{-1}$]
L3:Ni(II)	7.0	428[a]	41.6	473[a]	−0.38
				409[a]	0.11
				258[c]	0.86
	7.51	428[a]	106.7	473[a]	−1.15
				409[a]	0.44
				258[c]	2.47
	8.0	428[a]	121.7	473[a]	−1.36
				409[a]	0.50
				258[c]	−2.89
	8.62–10.06	427[a]	125.8–135.2	472[a]	−1.41
				409[a]	0.51
				258[c]	2.96
	10.52	425[a]	135.3	472[a]	−1.43
				409[a]	0.38
				259[c]	3.26
	11.0	425[a]	137.2	471[a]	−1.52
				409[a]	0.28
				259[c]	3.81

[a] d-d transition [b] $NH_2 \rightarrow Ni(II)$ CT [c] $N^- \rightarrow Ni(II)$ CT.
